# Research on the Trajectory Model for ZY-3

**DOI:** 10.1155/2014/429041

**Published:** 2014-08-27

**Authors:** Yifu Chen, Zhong Xie

**Affiliations:** National Engineering Research Center for Geographic Information System, China University of Geosciences, 388 Lumo Road, Wuhan 0086-430074, China

## Abstract

The new generation Chinese high-resolution three-line stereo-mapping satellite Ziyuan 3 (ZY-3) is equipped with three sensors (nadir, backward, and forward views). Its objective is to manufacture the 1 : 50000 topographic map and revise and update the 1 : 25000 topographic map. For the push-broom satellite, the interpolation accuracy of orbit and attitude determines directly the satellite's stereo-mapping accuracy and the position accuracy without ground control point. In this study, a new trajectory model is proposed for ZY-3 in this paper, according to researching and analyzing the orbit and attitude of ZY-3. Using the trajectory data set, the correction and accuracy of the new proposed trajectory are validated and compared with the other models, polynomial model (LPM), piecewise polynomial model (PPM), and Lagrange cubic polynomial model (LCPM). Meanwhile, the differential equation is derivate for the bundle block adjustment. Finally, the correction and practicability of piece-point with weight polynomial model for ZY-3 satellite are validated according to the experiment of geometric correction using the ZY-3 image and orbit and attitude data.

## 1. Introduction

Most high-resolution remote-sensing satellites are the near polar satellite; these satellites generally run on their trajectory below 1000 km in order to acquire the higher resolution for earth observation [1]. Ziyuan 3 (ZY-3) is the first Chinese civilian high-resolution stereo-mapping satellite that is equipped with three-line sensors (nadir, backward, and forward views) which have the separated optic system, respectively, and an additional multispectral sensor. The resolutions of nadir, backward, and forward views, are 2.5 m, 4.0 m, and 4.0 m, respectively, and the resolution of the multispectral sensor is 8 m [2]. The trajectory height of ZY-3 is relatively lower, 505 km. Thke satellite therefore is easily impacted by various disturbing forces from space and various flutters and jitters from the internal mechanical motion of the satellite such as high-frequency flutter from Gyro-Star and flywheel and the low-frequency jitter from solar panels. All of these factors result in the high-frequency flutter and low-frequency jitter of satellite when it is running on its trajectory [3].

For the linear push-broom satellite, every acquired image line has different data of orbit and attitude, and the instrument just records the data at regular intervals, but not all. The unrecorded data at a certain time therefore needs to be interpolated using exterior orientation model [4, 5]. For the traditional bundle block adjustment, it is almost impossible to acquire the solution using the orbit and attitude data of every image line. Thus, the high-precision exterior orientation model is needed to be researched and proposed, which is crucial for the geometry-data processing of linear push-broom satellite. In the geometry-data processing with block bundle adjustment, the difficult and key problem is how to reduce and eliminate the correlation between the interior and exterior orientation model parameters and improve the interpolation's accuracy of orbit and attitude, which avoid the transmission of the interpolation error to the interior orientation model in order to improve the solved accuracy of parameters [6, 7]. The interior and exterior model parameters will affect each other and make the cross-correlations in the calculation with bundle block adjustment. When the correlation among the parameters is strong, the systematic error cannot be described completely and accurately with these parameters, which result in the unstable oscillation of solved parameters and the decreased solution accuracy. In this process, the interpolation error of exterior elements (orbit and attitude) with trajectory model will also be transmitted to interior orientation as a part of systematic error, increase the systematic error of interior orientation, and decrease the solved accuracy and stability of geometric data processing. An ideal trajectory model not only can ensure a high interpolation accuracy for the attitude and orbit of every image line but also can decrease and eliminate the strong correlations among the solved parameters in bundle block adjustment and reduce the transmission of systematic error between the interior and exterior orientation models.

Generally, the satellite running trajectory is relatively stable in a short period so that the orbit and attitude in a short interval trajectory can be modeled with the polynomial, therefore avoiding the complex stress analysis of the satellite [8], therefore avoiding the complex stress analysis of the satellite. Currently, the trajectory models used for high-resolution remote-sensing satellite have polynomial model (LPM), piecewise polynomial model (PPM), and Lagrange cubic polynomial model proposed by Hofmann (LCPM) [9–11]. By research and comparison with the trajectory models, however, these models have some limitations. The LPM and PPM can acquire the smooth fitted curves, and the interpolation accuracies are very low especially for the unstably oscillated trajectory data. The LCPM can acquire a higher interpolation accuracy comparing with the LPM and PPM; however the LCPM easily causes the data oscillation, when the higher-order model is utilized in the block adjustment.

For satellite ZY-3, a new trajectory model (piece-point polynomial with weight model) is proposed to acquire the higher interpolation's accuracy in this paper, based on analyzing the orbit and attitude data of ZY-3. In addition, the data set of ZY-3 is used to validate the correction of piece-point with weight polynomial model and compare it with the other trajectory models, LPM, PPM, and LCPM, used for other satellites. Meanwhile, the differentiation equation of the proposed trajectory model is derivate and it is validated with the block bundle adjustment. According to geometric correction experiment with the different ground control points, the correctness and applicability of piece-point with weight polynomial model are validated and assessed to ensure and improve the high accuracy of geometric correction for ZY-3 satellite.

## 2. ZY-3 Trajectory Model

### 2.1. Piece-Point with Weight Polynomial Model

Trajectory model is a mathematic relationship elucidating the orbit and attitude of satellite vary with the different time in its track. For the push-broom satellite, every acquired image line has a different data of orbit and attitude, and the instrument just records the data at regular intervals, but not all the data. The unrecorded data at a certain time thereby needs to be interpolated using exterior orientation model.

Piece-point with weight polynomial model (PWPM) is proposed according to the researching and analyzing of the data of ZY-3's orbit and attitude in the long and short period. In comparison with the other models, LPM, PPM, and LCPM, the weight value is used in the new model to perform interpolation's calculation. The new model therefore can acquire a higher accuracy, have a better flexibility, and can reduce the correlation among the exterior orientation elements. The PWPM is an interpolation model with weight value. Using the model to interpolate satellite's orbit and attitude, the weight is calculated by the difference from any interpolation's time to the time assumed as known. Through the least square method, the polynomial parameters are solved, and then the exterior orientation at any time on the orbit can be acquired with the polynomial parameters. The PWPM is represented by (1), and the weight value is described with *P*:

(1) XSt=X0i+X1i·t+X2i·t2,YSt=Y0i+Y1i·t+Y2i·t2,ZSt=Z0i+Z1i·t+Z2i·t2,ϕt=ϕ0i+ϕ1i·t+ϕ2i·t2,ωt=ω0i+ω1i·t+ω2i·t2,κt=κ0i+κ1i·t+κ2i·t2,P=1||t−ti|| P=1(t−ti)2

**Figure 1:**
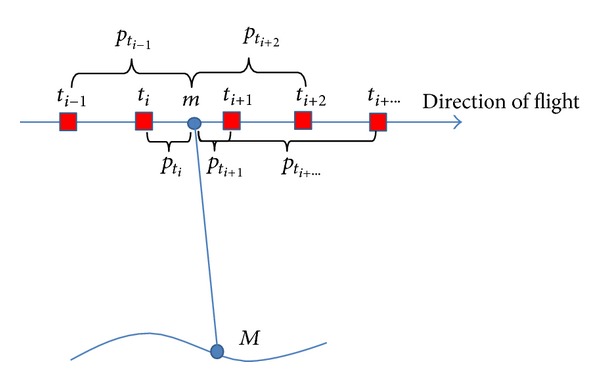
Diagram of piece-point with weight polynomial model.

Figure 1 is the diagram of PWPM. *M* is a ground point. Its imaging point is shown by *m*. *t* is the time that the point *m* is imaged. On the orbit, *t*_*i*−1_, *t*_*i*_, *t*_*i*+1_, *t*_*i*+2_, and *t*_*i*+⋯_ are the known times that the exterior orientations are acquired with GPS and star sensor, which are represented by red square. *p*_*i*−1_, *p*_*i*_, *p*_*i*+1_, *p*_*i*+2_, and *p*_*i*+⋯_, respectively, represent the weight of the time*t* to the known time *t*_*i*−1_, *t*_*i*_, *t*_*i*+1_, *t*_*i*+2_, and *t*_*i*+⋯_. The unrecorded orbit and attitude at every time like time *t* can be interpolated with the exterior orientations at the time *t*_*i*−1_, *t*_*i*_, *t*_*i*+1_, *t*_*i*+2_, and *t*_*i*+⋯_ using PWPM.

Assuming that the four known times *t*_1_, *t*_2_, *t*_3_ and *t*_4_ for orbit and attitude are selected as known points, the value of orbit and attitude at time t can be calculated and acquired with PWPM. The same polynomial coefficients are solved in the piece-wise polynomial model for the orbit and attitude respectively. For the convenient expression, the error equation is represented only with the angle Kappa as (3) according to (1) and the general form of error

(2) $$\begin{array}{c} V=A\cdot X-L \end{array}$$

(3) $$\begin{array}{c} v_{i}=\kappa_{0}^{i}+\kappa_{1}^{i}\cdot t+\kappa_{2}^{i}\cdot t^{2}-\kappa_{t}\;\left(i=1,2,3,4\right). \end{array}$$

Due to the four known times, the four error equations can be established and the coefficient matrix *A*, observed value matrix *L*, and unknown vector matrix *X* are constructed by error equations. Matrices *A*, *L*, and *X* are represented by

(4) $$\begin{array}{c} A=\left[\begin{array}{ccc} 1 & t_{1} & t_{1}^{2}\\ 1 & t_{2} & t_{2}^{2}\\ 1 & t_{3} & t_{3}^{2}\\ 1 & t_{4} & t_{4}^{2} \end{array}\right];\;\;L=\left[\begin{array}{c} \kappa_{1}\\ \kappa_{2}\\ \kappa_{3}\\ \kappa_{4} \end{array}\right];\;\;X=\left[\begin{array}{c} k_{0}\\ k_{1}\\ k_{2} \end{array}\right]. \end{array}$$

According to the equation of weight, the weights *p*_1_, *p*_2_, *p*_3_, and *p*_4_ for times *t*_1_, *t*_2_, *t*_3_, and *t*_4_, respectively, are calculated and shown in (5), and then the weight matrix *P* is established. The value of weight reflects the influence degree of the orbit and attitude at times *t*_1_, *t*_2_, *t*_3_, and *t*_4_ for the unknown time *t*:

(5) p1=1(t−t1)2;  p2=1(t−t2)2;p3=1(t−t3)2;  p4=1(t−t4)2(6)P=[p1p2p3p4].

According to the least square method, normal equation coefficient matrix *N* and free vector *U* are constructed and shown by

(7) N=ATPA=[p1+⋯+p4p1t1+⋯+p4t4p1t12+⋯+p4t42p1t1+⋯+p4t4p1t12+⋯+p4t42p1t13+⋯+p4t43p1t12+⋯+p4t42p1t13+⋯+p4t43p1t14+⋯+p4t44]U=[κ1·p1+⋯+κ4·p4κ1·t1·p1+⋯+κ4·t4·p4κ1·t12·p1+⋯+κ4·t42·p4].

For convenience, the matrices *N* ^−1^ and *U* are represented by the below formation, shown in

(8) $$\begin{array}{c} N^{-1}=\left[\begin{array}{ccc} c_{1} & c_{2} & c_{3}\\ c_{4} & c_{5} & c_{6}\\ c_{7} & c_{8} & c_{9} \end{array}\right]\;\;U=\left[\begin{array}{c} u_{1}\\ u_{2}\\ u_{3} \end{array}\right]. \end{array}$$

Through least square adjustment, the polynomial parameters *k*_0_, *k*_1_, and *k*_2_ are solved and shown by

(9) $$\begin{array}{c} X=\left[\begin{array}{c} k_{0}\\ k_{1}\\ k_{2} \end{array}\right]=\left[\begin{array}{ccc} c_{1} & c_{2} & c_{3}\\ c_{4} & c_{5} & c_{6}\\ c_{7} & c_{8} & c_{9} \end{array}\right]\cdot \left[\begin{array}{c} u_{1}\\ u_{2}\\ u_{3} \end{array}\right]. \end{array}$$

Afterwards, the Kappa value at *t* time can be calculated using (1), and the general formula of the Kappa value is represented by

(10) κ(t)  =(c1u1+c2u2+c3u3) +(c4u1+c5u2+c6u3)t +(c7u1+c8u2+c9u3)t2.

In the process of interpolation, the PWPM can solve the different parameters corresponding to the different attitude and orbit at any time with the different weight value. In this paper, the two weight equations are given out, the reciprocal of the absolute value of time difference and the reciprocal of the square of the time difference. The selection of weight equation has a large impact for the interpolation's accuracy of orbit and attitude. According to analysis and research, the reciprocal of the square of the time difference is adopted when the trajectory is relatively unstable. On the contrary, the reciprocal of the absolute value of time difference is utilized.

### 2.2. Two Interpolation Methods of Trajectory Model

For the PWPM, the new trajectory has two kinds of interpolation methods to acquire the data set of orbit and attitude at any time. One is using the several known times selected round the unknown time to interpolate the orbit and attitude at the unknown time. The other is using all the selected known times to interpolate the orbit and attitude at the unknown time. The impaction from the selected known time for the orbit and attitude at the unknown time is measured and assessed according to the weight value. In other words, the time difference between the unknown time and the known time is more far, and the impaction from the unknown time is more great. The interpolation accuracies with the two methods are different, which is determined by the stability of the satellite trajectory, the number of selected known times, and the location of the selected known time. In the practical application, the two methods of PWPM are utilized together or respectively, which is determined by the stability of orbit and attitude.

### 2.3. The Differential Expression of PWPM

Sensor's imaging model describes the mathematic transformation relationship between the coordinate of image point (*x*, *y*) and the coordinate of ground point (*X*, *Y*, *Z*). It includes two kinds of models, rigorous imaging model and general imaging model [12, 13]. Based on the structure of CCD-array equipped on the ZY-3 and the sight vector of every CCD, scanning the ground, the rigorous imaging model for ZY-3 satellite is established, represented by (11), and the one to one correspondence relationship between image point and ground point is built up with sensors coordinate systems, satellite's trajectory coordinate system, and ground reference system.

For ZY-3 satellite, the data received from dual-frequency GPS represents the location of the phase center of GPS and the attitude data from star sensor is measured in the J2000 coordinate [14, 15]. In the process, the displacement matrix from the phase center of GPS (GPS antenna) to the coordinate of satellite body, ${\left[\begin{array}{ccc} Dx & Dy & Dz \end{array}\right]}^{T}$; the displacement matrix from CCD-array center to the coordinate of satellite body, ${\left[\begin{array}{ccc} dx & dy & dz \end{array}\right]}^{T}$; the rotation matrix from the coordinate of star sensor to the coordinate of satellite body, *R*_star_^body^; and the rotation matrix form imaging space coordinate to the coordinate satellite body, *R*_camera_^body^, are needed:

(11) [XYZ]WGS84=m·R·[[DxDyDz]+[dxdydz]+Rcamerabody·(xy−f)] +[XYZ]GPSR=RJ2000WGS84·RorbitJ2000·Rbodyorbit,

where ${\left[\begin{array}{ccc} x & y & -f \end{array}\right]}_{}^{T}$ are point coordinates in the image system; ${\left[\begin{array}{ccc} X & Y & Z \end{array}\right]}_{\text{GPS}}^{T}$ and ${\left[\begin{array}{ccc} X & Y & Z \end{array}\right]}_{\text{WG}{\text{S}}_{84}}^{T}$ are perspective center position and position coordinates in WGS84 coordinate system; *R*_body_^orbit^, *R*_orbit_^J2000^, and *R*_orbit_^J2000^ are rotation matrices, respectively, from satellite body system to satellite orbit system, from satellite orbit system to J2000 coordinate system, and from J2000 coordinate system to WGS84 coordinate system; *m* represents the scale. According to satellite's structure design and the result of laboratory calibration, the three-line imaging model, nadir, forward, and backward, can be acquired with the different value of displacement matrix ${\left[\begin{array}{ccc} dx & dy & dz \end{array}\right]}^{T}$ and rotation matrix *R*_camera_^body^.

For the push-broom high-resolution satellite, the objective of the high-accuracy trajectory model is to acquire the accurate elements of exterior orientation, [*X*_GPS_, *Y*_GPS_, *Z*_GPS_]^*T*^ and *R*_body_^orbit^, at any time.

Assuming that the n known times are selected in a scene image of satellite and the orbit and attitude at time *t* are needed to be interpolated, the four known times *t*_1_, *t*_2_, *t*_3_, and *t*_4_ round the unknown time *t* are selected and the their weight values *p*_1_, *p*_2_, *p*_3_, and *p*_4_ are correspondingly calculated with (1). For the convenient expression, the Pitch angle is picked up as a sample; therefore, the value of Pitch angle at *t* time can be calculated and represented by (12), which has the same form expression as (10):

(12) Pitch(t)=(c1u1+c2u2+c3u3)+(c4u1+c5u2+c6u3)t+(c7u1+c8u2+c9u3)t2.

In the calculation of bundle block adjustment, the differential expression of *t* time for the four unknown times *t*_1_, *t*_2_, *t*_3_, and *t*_4_ is derived and represented by

(13) ∂Pitch(t)∂Pitch(t1)=(c1+c4t+c7t2)p1+(c2+c5t+c8t2)p1t1+(c3+c6t+c9t2)p1t12∂Pitch(t)∂Pitch(t2)=(c1+c4t+c7t2)p2+(c2+c5t+c8t2)p2t2+(c3+c6t+c9t2)p2t22∂Pitch(t)∂Pitch(t3)=(c1+c4t+c7t2)p3+(c2+c5t+c8t2)p3t3+(c3+c6t+c9t2)p3t32∂Pitch(t)∂Pitch(t4)=(c1+c4t+c7t2)p4+(c2+c5t+c8t2)p4t4+(c3+c6t+c9t2)p4t42.

Similarly, the differential expression of the other elements of exterior orientation, (Roll, Yaw) and (*X*_*s*_, *Y*_*s*_, *Z*_*s*_), can be derived.

## 3. Systematic Error Model 

The systematic error model for the interior orientation is to describe the various distortions from satellite's sensor such as the CCD-array distortions, the distortions of optic lenses, and principal point's distortion. In order to realize the high-precision geometric correction for ZY-3 image, it is very necessary to establish the various error models based on the analysis of satellite's structural parameters; thus the system error coming from the interior orientation, radial direction, and tangential direction distortion of optics lens and CCD-line's distortion and rotation will be modeled [16, 17]. According to the analysis of satellite's structure and the imaging characteristics, the systematic error model of the interior orientation is established and represented by

(14) Δx=−Δffx−+(k1r2+k2r4)x−   +p1(r2+2x−2)+2p2x−y−+y−sinθΔy=−Δffy−+(k1r2+k2r4)y−   +p2(r2+2y−2)+2p1x−y−+syy−x¯=(x−x0), y¯=(y−y0), r=x−2+y−2,

where $\left(-\Delta f/f\right)\overset{-}{x}$ and $\left(-\Delta f/f\right)\overset{-}{y}$ represent the errors generated by the image principal point and focal length; Δ*f* and *f* mean the difference of focal length and the optic focal length, respectively; $(k_{1}r^{2}+k_{2}r^{4})\overset{-}{x}$ and $(k_{1}r^{2}+k_{2}r^{4})\overset{-}{y}$ describe the optics lens distortion of radial direction in along-track and cross-track directions, respectively; *k*_1_, *k*_2_ mean the distortion's coefficient of radial direction; *r* means the distance from one point on the optic lens to the lens's center; $p_{1}(r^{2}+2{\overset{-}{x}}^{2})+2p_{2}\overset{-}{x}\overset{-}{y}$ and $p_{2}(r^{2}+2{\overset{-}{y}}^{2})$ represent the distortions of tangential direction in along-track and cross-track directions, respectively; *p*_1_, *p*_2_ mean the distortion's coefficient of tangential direction; $\overset{-}{y}\sin\theta$ represents the error of CC-array rotation, and *θ* is the rotation angle; $s_{y}\overset{-}{y}$ represents the distortion of CCD-array in the cross-track direction generated by the temperature variation. The CCD-array distortion in along-track direction is particle, owing to only one CCD arranged in this direction; the distortion therefore can be ignored.

According to the analysis of the correlations among the model's parameters in the block bundle adjustment, the correlation between the principal point and focal length is very strong, so that the parameters are combined in order to reduce the parameters correlation and improve the stability and accuracy of the block bundle adjustment. Equation (14) is represented as (15) after the parameters combination:

(15) Δx=x0+(k1r2+k2r4)x−+p1(r2+2x−2)   +2p2x−y−+y−sinθΔy=y0+(k1r2+k2r4)y−+p2(r2+2y−2)   +2p1x−y−+syy−x¯=(x−x0), y¯=(y−y0), r=x−2+y−2.

## 4. Data and Method of Experiment

In this paper, data set of orbit and attitude used to validate the correction and accuracy of piece-point polynomial model is acquired from 609th track of ZY-3. In order to validate the high accuracy of the new proposed trajectory model, the LPM, PPM, LCPM, piece-point with weight polynomial with four known times model (PWP4M) and piece-point with weight polynomial model with all known times (PWPM) are utilized to interpolate and compare the interpolation accuracy. In the process, the different numbers of the known times, 10, 15 and 20, are selected from trajectory data set, and are used to interpolate the other unknown times' orbit and attitude data with the different models, respectively. Finally, the result of interpolation is represented by the table and curve, and the advantage of PWPM is illuminated according to researching and analyzing the result.

**Figure 2:**
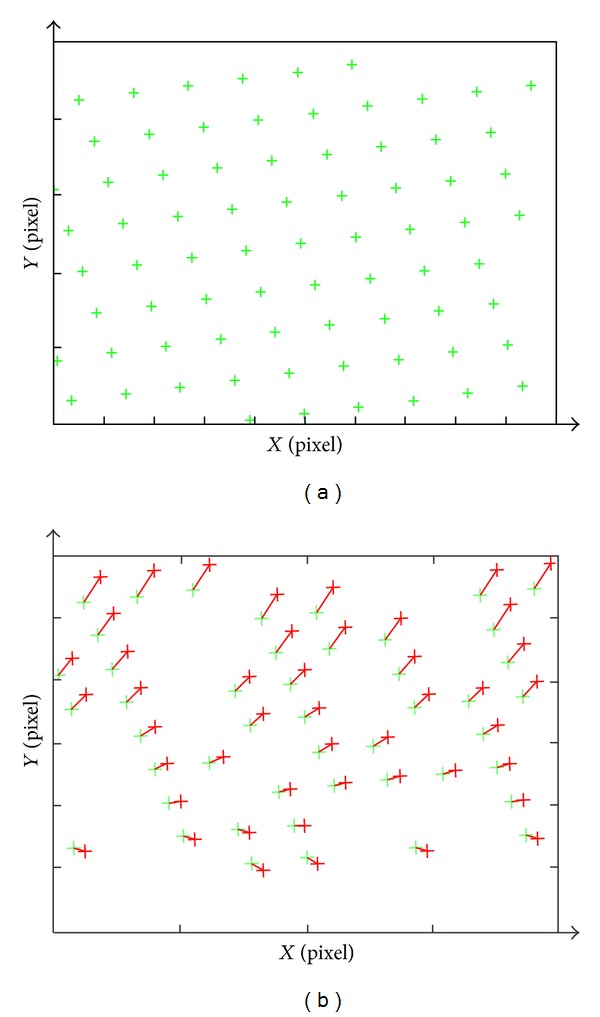
(a) Diagram of the distribution of GCPs; (b) the error's distribution of image points corresponding to GCPs.

In order to validate the correction and accuracy of PWPM, ZY-3 orbit, and attitude data, ground control point (GCP) and systematic error model of interior orientation are used in the bundle block adjustment of geometric correction. Based on the nadir image of ZY-3, the 74 GCPs are picked up from the image, and 27 GCPs are selected as check points (CPs) that do not take part in the block bundle adjustment. For validating the correction and stability of the proposed models, the 16, 26, 36, and 46 GCPs are performed, respectively, in the geometric correction experiment.  Figure 2 shows the distribution of GCPs and the error's distribution of image points, corresponding to GCPs, before geometric correction.

## 5. Result and Validation of Experiment

### 5.1. Result and Analysis of Trajectory Model

**Figure 3:**
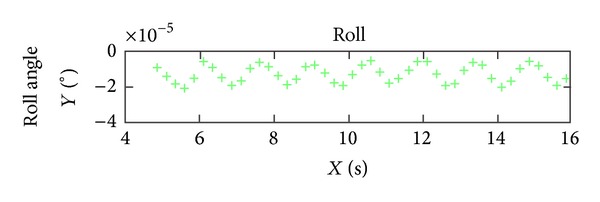
The attitude angle (Roll) curve in 10 seconds.

**Figure 4:**
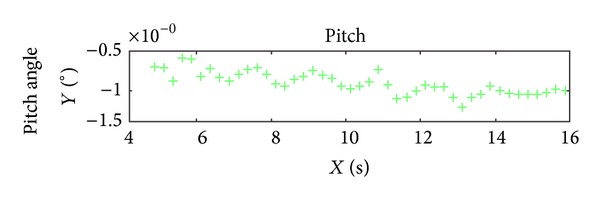
The attitude angle (Pitch) curve in 10 seconds.

**Figure 5:**
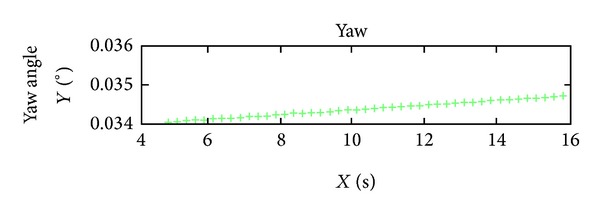
The attitude angle (Yaw) curve in 10 seconds.

According to analyzing the stability of the orbit and attitude of ZY-3, it can be seen obviously that the orbit and attitude angles of Yaw of ZY-3 are very stable, but the attitude angles of Pitch and Roll are unstable relatively. The curves of attitude angles in 10 seconds are shown in Figures 3, 4, and 5. From the diagram of curves, it is obvious that the attitude angles of Pitch and Roll are unstable. Hence, the interpolation experiment is performed using the angle Pitch and Roll.

**Figure 6:**
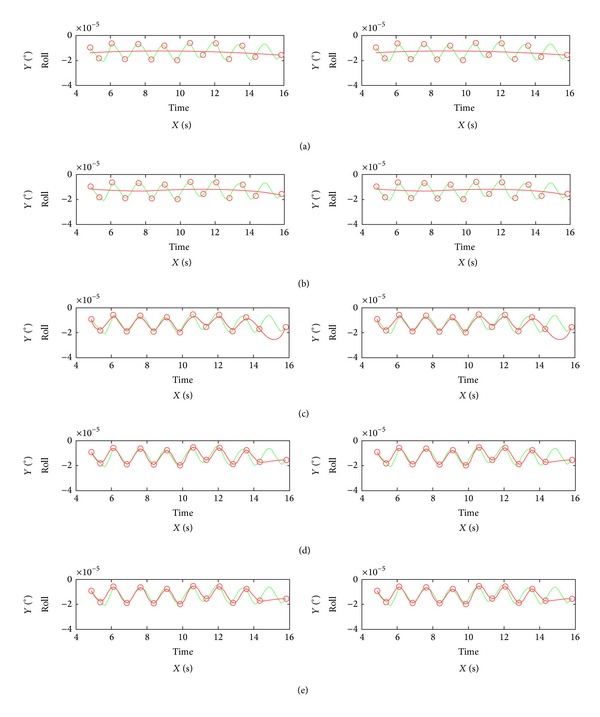
The fitting curves with the different trajectory models.

In Figure 6, the result of interpolation is represented using the different trajectory models with 15 selected known times in a scene image. The curves on the left of Figure 6 show the interpolation's result with angle Pitch, and the curves on the right of Figure 6 show the result with angle Yaw. The red curve and red circle, respectively, mean the fitting curve and the selected known times, and the green curve means the original curves. From top to bottom Figures 6(a), 6(b), 6(c), 6(d), and 6(e) represented, respectively, the models LPM, PPM, LCPM, PWP4M, and PWPM.

From Figure 6, it can be seen clearly that the interpolation's accuracy with LCPM, PWP4M, and PWPM is higher than LPM and PPM. The curves of LCPM, PWP4M, and PWPM are relatively similar. Furthermore, Table 1 shows the interpolation accuracy results with the different orbit and attitude models using the different numbers of known times selected from 609th track orbit in a scene image. The accuracy of the interpolation for the angles Roll, Pitch, and Yaw with PWPM is the highest with 10 and 15 selected known times. Using the 20 known times, the interpolation accuracy of Yaw is the highest with PWP4M, 4.539. The accuracy of Pitch with PWP4M is 5.727 lower than the accuracies with PWPM 5.593. In this case, PWP4M and PWPM can be used together in order to acquire the highest accuracy of interpolation for any angle.

### 5.2. Result and Analysis of Geometric Correction Based on PWPM

**Figure 7:**
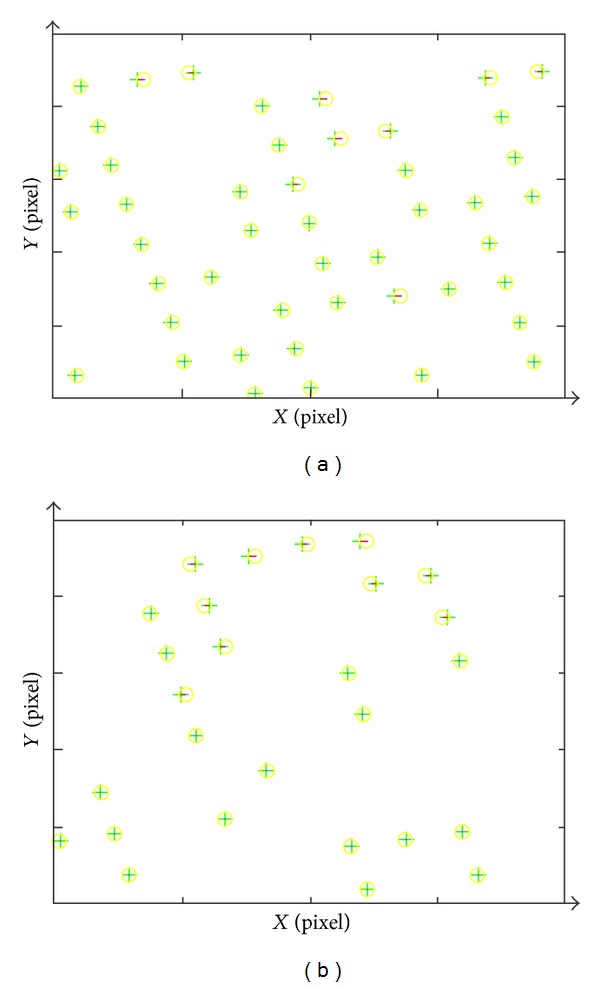
Diagram of geometric correction: (a) the residuals of GCP; (b) assessment with CP.

The new proposed trajectory model (PWPM) has higher interpolation's accuracy and more flexibility than the other models according to the upper experiment and analysis. In order to validate the correction and accuracy of PWPM in the bundle block adjustment, the geometric correction experiment is performed using the data set of ZY-3. Before the process, which one interpolation's method of PWPM is utilized according to the analysis of the orbit and attitude corresponding to the used image range? Thus, geometric correction is performed and the result of correction is represented by Figure 7.

In Figure 7(a), the residuals distribution of 46 GCPs after the geometric correction is represented and the residuals distribution after checking with 27 CPs is shown in Figure 7(b) and the assessed accuracy is 0.0793 pixels. From Figure 7, it is obvious that the accuracy of geometric correction is very high based on the PWPM. Furthermore, Table 2 shows the assessment of accuracy for geometric correction using the 10, 16, 26, 36, and 46 GCPs, respectively. The accuracies with the different number of GCPs are all high; the highest accuracy reaches 0.5293 pixels with 26 GCPs and the lowest accuracy is 0.0841 pixels. With the increasing number of GCPs, the accuracy of geometric correction increased gradually until the 26 GCPs.

### 5.3. Validation and Analysis

Analyzing and comparing Table 1 and Figure 6, it is obvious that the interpolation accuracy of PWPM is the highest. When the orbit and attitude are unstable, the weight value is acquired with the reciprocal value of the absolute value of time difference. On the contrary, the weight value is calculated with the reciprocal value of the square of time difference. In comparison with the LPM, PPM, and LCPM, PWPM can solve the different parameters corresponding to the different attitude at any time with the different weight value. The proposed new trajectory model can therefore reach higher accuracy of interpolation than others, especially when the orbit and attitude are unstable. In addition, the PWPM has two interpolation methods, PWP4M and PWPM, and the two methods can be used together in order to acquire the higher interpolation accuracy. Owing to the higher accuracy, the new trajectory model has the ability that can improve the interpolation accuracy of orbit and attitude and avoid the interpolation error is transmitted into interior orientation as a part of systematic error, which will increase the systematic error of interior orientation. Thus, the solved accuracy of parameters in the systematic model is improved, and the accuracy of geometric correction is also increased correspondingly. According to the analysis and research, the different form of PWPM in the bundle block adjustment only relates to weight, selected known time, and needed interpolation time; thus the correlation among the orbit and attitude is decreased, and the calculation's accuracy and stability of the block adjustment can be improved.

In the geometric correction experiment based on the PWPM, the accuracies with the different number of GCPs also reach a high level totally, which is represented by Table 2 and Figure 7, and validate the correctness and applicability of the PWPM. For the different number of GCPs, the accuracy varies in the geometric correction mostly owing to two reasons, the distribution of the different GCPs and the correlations among the parameters of the systematic error model. On the one hand, the various distributions of the different GCPs will cause the accuracy to oscillate in a very small range; on the other hand, the bundle block adjustment will generate correlations among the parameters. The correlations result in the following: the solved results of parameters vibrate in a range unstably, and the systematic error cannot be described completely and accurately with these parameters. Thus, a better systematic error model is needed to be proposed according to further researching and analyzing of the satellite sensor's overall structure design and the imaging geometric characteristics.

## 6. Conclusion 

In this study, the new trajectory model, PWPM, is proposed according to the researching and analyzing of the data of ZY-3's orbit and attitude in the long and short period. By comparison with the other trajectory models, the PWPM can acquire a higher interpolation's accuracy and has more flexibility. Meanwhile, the differentiation equation of the proposed trajectory model is derivate and it is validated through the bundle block adjustment. In the geometric correction experiment based on the PWPM, the accuracies of geometric correction with the different number of GCPs also reach a high level totally. According to the analyzing and researching of the assessment results with GCPs and CPs, the correctness and applicability of the PWPM are validated and assessed to ensure and improve the high accuracy of geometric correction for ZY-3 satellite. The further study will be performed to experiment with the real image data of ZY-3 and GCP to research better systematic error model for interior orientation, in order to explore the potentials of using ZY-3 data for stereo mapping.

## Figures and Tables

**Table 1:** The fitting accuracy comparison of the different attitude and orbit modes selecting the different known times on the orbit (unit: degree).

*σ* (*e* − 007)	10			15			20		
Angle	Roll	Pitch	Yaw	Roll	Pitch	Yaw	Roll	Pitch	Yaw
1-LPM	64.610	12.772	15.100	47.859	9.103	15.297	47.362	8.569	15.122
2-PPM	63.136	12.237	16.210	47.768	8.737	15.027	47.281	8.532	14.948
3-LCPM	63.523	11.955	13.919	51.758	8.275	10.257	45.901	5.946	9.554
4-PWP4M	63.668	11.732	13.956	34.760	8.034	7.7186	15.628	5.727	4.539
5-PWPM	60.287	10.387	12.534	32.837	7.648	7.5958	22.261	5.593	5.140

**Table 2:** The assessment of accuracy for geometric correction (unit: pixel).

Number of GCPs	σ_*x*_	σ_*y*_	σ_sum_
46	0.0767	0.0215	0.0797
36	0.0706	0.0217	0.0739
26	0.0508	0.0146	0.0529
16	0.0725	0.0161	0.0743
10	0.0795	0.0276	0.0841
